# Histone deacetylase-mediated regulation of the antimicrobial peptide hBD2 differs in intestinal cell lines and cultured tissue

**DOI:** 10.1038/s41598-018-31125-x

**Published:** 2018-08-27

**Authors:** Sabrina Stebe-Frick, Maureen J. Ostaff, Eduard F. Stange, Nisar P. Malek, Jan Wehkamp

**Affiliations:** 1 Department of Hepatology, Gastroenterology and Infectiology, University Hospital, 72076 Tübingen, Germany; 2 Dr. Margarete Fischer-Bosch Institute of Clinical Pharmacology and University of Tübingen, 70376 Stuttgart, Germany; 3 Present Address: Scientific Affairs - Philips Image guided therapy devices, Colorado Springs, Colorado USA

## Abstract

Histone deacetylase inhibition (HDACi) has been suggested as a promising approach to bolster TLR-mediated induction of antimicrobial peptides such as human β-defensin 2 (hBD2). In inflammatory bowel disease (IBD), Crohn’s disease (CD) patients display an attenuated expression of hBD2 as compared to ulcerative colitis (UC). Here, we aimed to study if combining HDACi with the therapeutic *E. coli* Nissle 1917 (EcN), a strong hBD2 inducer, might be a feasible strategy to further modify protective immune responses. Monolayer epithelial cell lines versus cultured human biopsies from healthy controls and CD and UC patients showed diverse effects. In mono-cell systems, we observed a strong NF-kB-dependent enhancement of TLR- but also IL1β-mediated hBD2 induction after HDACi. In contrast, multicellular colonic biopsy culture showed the opposite result and HDACi was associated with an abolished TLR-mediated hBD2 induction in all tested patient groups. Of note, CD patients showed an attenuated induction of hBD2 by *E. coli* Nissle as compared to UC. We conclude that the role of HDACs in hBD2 regulation is context-dependent and likely modified by different cell types. Differential induction in different IBD entities suggests different clinical response patterns based on still unknown hBD2-associated mechanisms.

## Introduction

Histone deacetylases (HDACs) are increasingly recognized as key epigenetic factors in regulating tissue homeostasis. By deactylating histones, they decrease DNA accessibility for transcription factors and thereby affect gene expression^1,2^. In addition, they have been shown to modify the acetylation status and consequently function of non-histone proteins such as nuclear factor κ-light-chain-enhancer of activated B cells (NF-κB)^3,4^. Their role in health and disease is intensively studied. Since the recent FDA approval of the first HDAC inhibitor (Vorinostat) to treat cutaneous T-cell lymphoma, other inhibitory compounds have been proposed for the treatment of other cancers but also disorders such as inflammatory bowel diseases (IBD)^5,6^.

In IBD, HDAC inhibitors may act through their regulatory function on human β-defensin 2 (hBD2). This is an inducible innate antimicrobial effector present in epithelia^7,8^, but also in immune cells^9–11^. It is upregulated in response to microbial and/or inflammatory stimuli and has a crucial role in defending the host against infectious threats. Interestingly, hBD2 has been shown to be upregulated in colonic epithelia in response to probiotic bacteria^12,13^, which is one of the proposed mechanisms by which probiotics bolster gut barrier function. The induction of protective hBD2 could at least in part explain these beneficial effects in remission maintenance of ulcerative colitis (UC)^14^. In the second IBD entity, Crohn’s disease (CD), the induction of β-defensins appears to be defective^15,16^ and this could explain the lacking probiotic benefit in these patients.

Here, we aimed to study relevant mechanisms that underlie the role of HDACs in hBD2 inducibility. Only a few years ago, Yin and Chung implicated HDACs as important regulators of the Toll like receptor (TLR)-dependent induction of hBD2 in gingival epithelial cells^17^. Besides these interesting findings, *in vitro* experiments could also show that dietary compounds with inhibitory effects on histone deacetylation processes moderate hBD2 induction in intestinal epithelial cell lines^18^. In addition, a recent report by Fischer *et al*.^19^ utilized colonic epithelial cell culture as well as primary cell organoids to highlight a role of HDACs in hBD2 regulation. These data showed that histone deacetylase inhibition can enhance hBD2 expression in response to *E. coli* K12^19^. Since K12 and other *E. coli* usually do only induce hBD2 to a slight extend and the induction is much stronger with probiotic strains such as *E. coli* Nissle 1917 (EcN)^13^, we studied the known probiotic EcN as a potent inducer of this defensin. In addition, we used human colonic biopsy culture to better reflect the *in vivo* situation. We have recently utilized a similar setting to study small intestinal innate immune regulation^20^ and found that cultured, freshly obtained biopsies can be a relevant model that is sometimes helpful in elucidating the more complex situation in a tissue context. While our monolayer data are consistent with the already described enhanced hBD2 induction following HDAC inhibition (HDACi), the opposite effect is observed in cultured biopsies. Based on the known differences in hBD2 expression between CD and UC patients^16^, we hypothesized that CD colonic biopsies would also show diminished hBD2 induction levels after *ex vivo* stimulation with EcN as compared to UC specimens. Overall, we aimed to gain a better understanding of the epigenetic regulation of the antimicrobial peptide hBD2 in different experimental settings inclduding *ex vivo* analysis of patient tissue culture.

## Results

### *In vitro* induction of human β-defensin 2 is strongly enhanced by histone deacetylase inhibition

**Figure 1:**
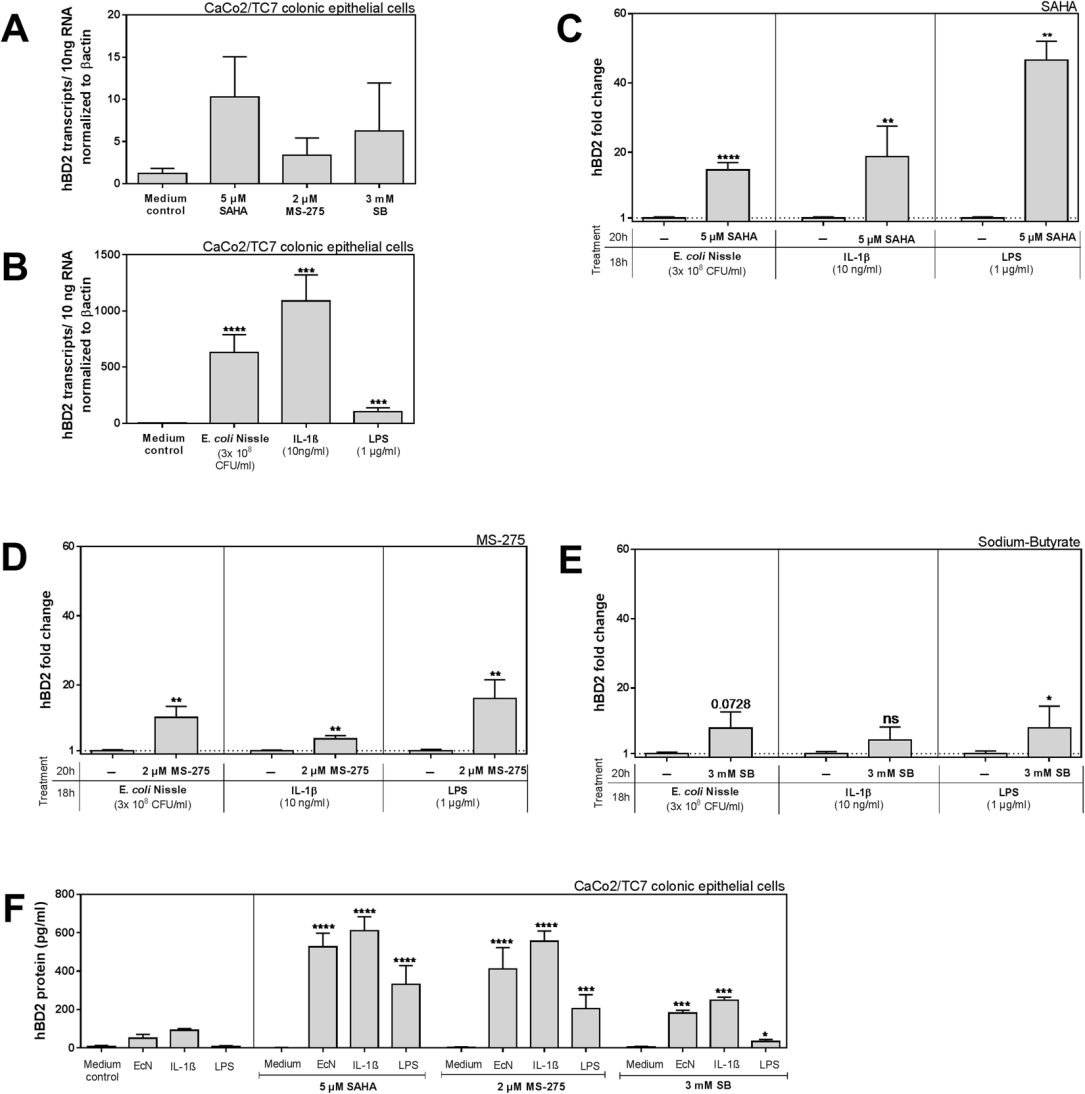
Enhancing effect of histone deacetylase (HDAC) inhibition on the induction of human β-defensin 2 (hBD2) in CaCo-2/TC7 cells in response to *E. coli* Nissle 1917 (EcN), Interleukin 1β (IL-1β) and lipopolysaccharide (LPS). HBD2 mRNA expression after (**A**) treatment with HDAC inhibitors SAHA (5 µM), MS-275 (2 µM) or SB (3 mM) each for 20 hrs or (**B**) with the probiotic strain *E. coli* Nissle 1917 (3 × 10^8^ CFU/ml), IL1β (10 ng/ml) or LPS (1 µg/ml) each for 18 hrs. Shown are relative copy numbers of hBD2 per 10 ng total RNA normalized to βactin expression. (**C–E**) Shows the fold change in hBD2 induction compared to each stimulation alone when co-treated with either SAHA (5 µM) (**C**), MS-275 (2 µM) (**D**) or SB (3 mM) (**E**). Here inhibitor treatment started 2 hrs prior to the stimulations, which took then place in parallel to HDAC inhibition for another 18 hrs. Shown are results of at least three independent experiments carried out in biological triplicates. *p < 0.05, **p < 0.01, ****p < 0.0001 evaluated by Mann-Whitney *u* test. (**F**) HBD2 peptide levels from CaCo-2/TC7 cell lysates as determined by ELISA. Treatment was carried out exactly as for mRNA analyses. Results represent three independent experiments measured as technical triplicates. *p < 0.05, ***p < 0.001, ****p < 0.0001 evaluated by Mann-Whitney *u* test.

We used the human colonic epithelial CaCo-2 subclone TC7 cells (CaCo-2/TC7) to study the role of different hBD2 stimuli (heat-inactivated EcN, lipopolysaccharide (LPS) and Interleukin 1β (IL1β)) without and with simultaneous inhibition of HDAC function by different compounds (suberoylanilide hydroxamid acid (SAHA), Pyridin-3-ylmethyl *N*-[[4-[(2-aminophenyl)carbamoyl]phenyl]methyl] carbamate (MS-275), or sodium-butyrate (SB)) (Fig. 1). The inhibitors alone did not have any effect within 20 hrs (Fig. 1A). Very low transcript numbers of hBD2 per 10 ng total RNA (40 transcripts max) can be considered negligible. Challenging the cells with the stimulants for 18 hrs however, induced high basal levels of hBD2 as shown in Fig. 1B. Strikingly, we detected a strong enhancement of the hBD2 levels as shown in Fig. 1B, when HDAC function was inhibited in parallel (Fig. 1C–E). This was especially true for the pan-HDAC-inhibitor SAHA (Fig. 1C). Using the more specific MS-275, which mainly blocks HDACs 1 and 3 at the deployed concentration^21–24^ (Fig. 1D), led to a comparably strong effect when combined with EcN, while for IL1β and LPS the trend was still significant but weaker. The naturally occurring short chain fatty acid derivate butyrate with known HDAC inhibitory function also augmented hBD2 induction albeit to a much smaller extent (Fig. 1E). This response in hBD2 expression could also be seen on the hBD2 protein level (Fig. 1F). In contrast the constitutively expressed human β-defensin 1 (hBD1) was not significantly affected by HDAC inhibition (Fig. S1A–C). Taken together, these results demonstrate an important role for HDACs in the transcriptional regulation of hBD2 in intestinal epithelial cells to the extent that they seem to perpetuate the level of hBD2 inducibility. Our observation is in line with a recent study demonstrating a similar effect in CaCo-2/TC7 cells and human colonic organoids using the inhibitors TSA and SAHA^19^.

**Figure 2:**
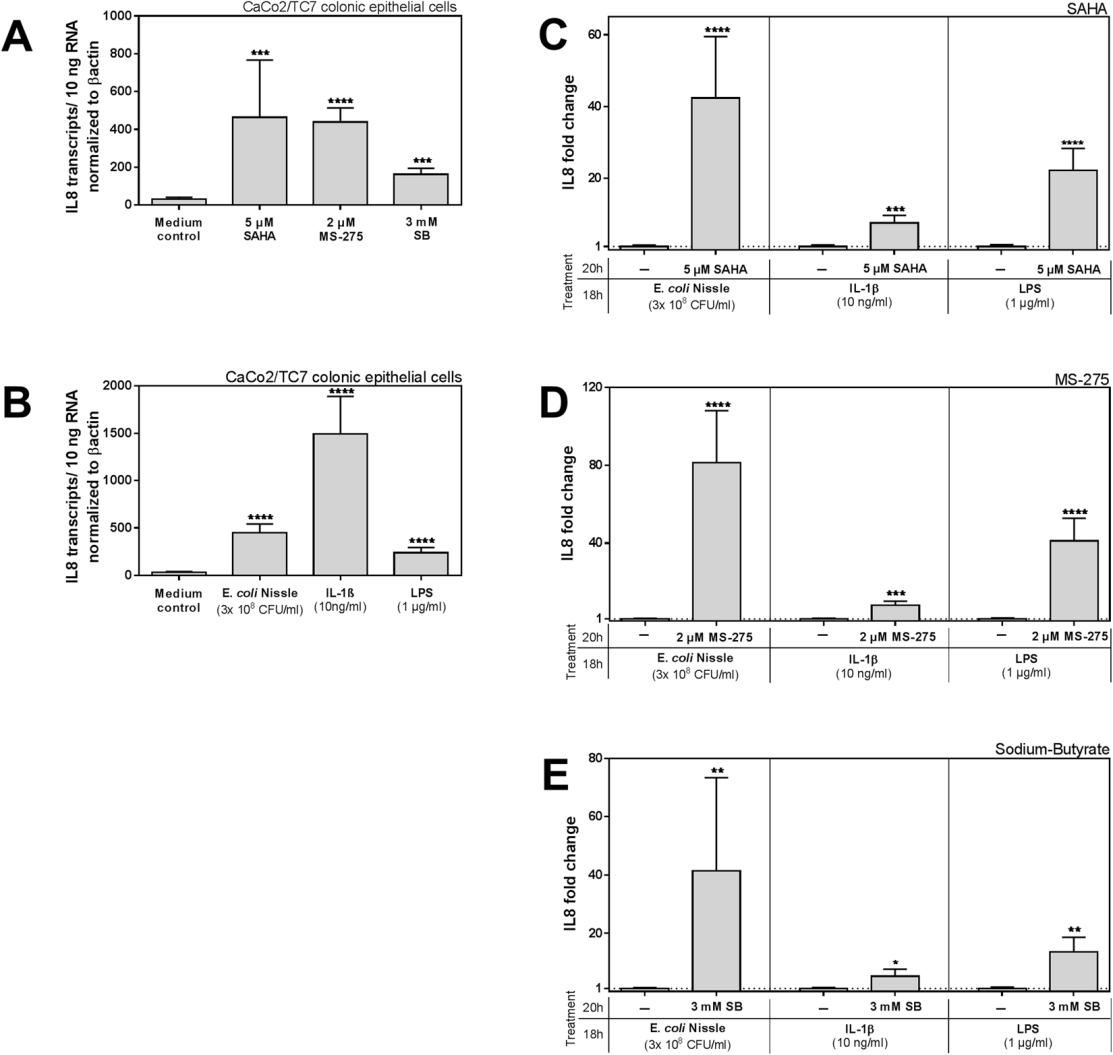
Effect of histone deacetylase (HDAC) inhibition on Interleukin 8 (IL8) expression in CaCo-2/TC7 cells. Expression of IL8 mRNA after (**A**) treatment with HDAC inhibitors SAHA (5 µM), MS-275 (2 µM) or SB (3 mM) each for 20 hrs or (**B**) with the probiotic strain *E. coli* Nissle 1917 (3 × 10^8^ CFU/ml), IL1β (10 ng/ml) or LPS (1 µg/ml) each for 18 hrs. Shown are relative copy numbers of hBD2 per 10 ng total RNA normalized to βactin expression. (**C**–**E**) Shows the fold change in IL8 induction compared to each stimulation alone when co-treated with either SAHA (5 µM) (**C**) MS-275 (2 µM) (**D**) or SB (3 mM) (**E**). Inhibitor treatment started 2 hrs prior to the stimulations, which took then place in parallel to HDAC inhibition for another 18 hrs. Shown are results of at least three independent experiments carried out in biological triplicates. *p < 0.05, **p < 0.01, ***p < 0.001, ****p < 0.0001 evaluated by Mann-Whitney *u* test.

To see if HDACi also influences the inflammatory response in CaCo-2/TC7 cells, we measured the expression of interleukin 8 (IL8) (Fig. 2). Interestingly, this pro-inflammatory cytokine responded to treatment with inhibitors alone, showing a significant induction (Fig. 2A). Figure 2B displays the basal induction levels of IL8 as mediated by the applied stimulants, which are similar to what we saw with hBD2, but slightly contrary to the previous report from Fischer and colleagues^19^. Simultaneous HDAC inhibition together with the stimulation acted synergistically on IL8 induction and strongly enhanced the expression of this pro-inflammatory gene (Fig. 2C–E).

**Figure 3:**
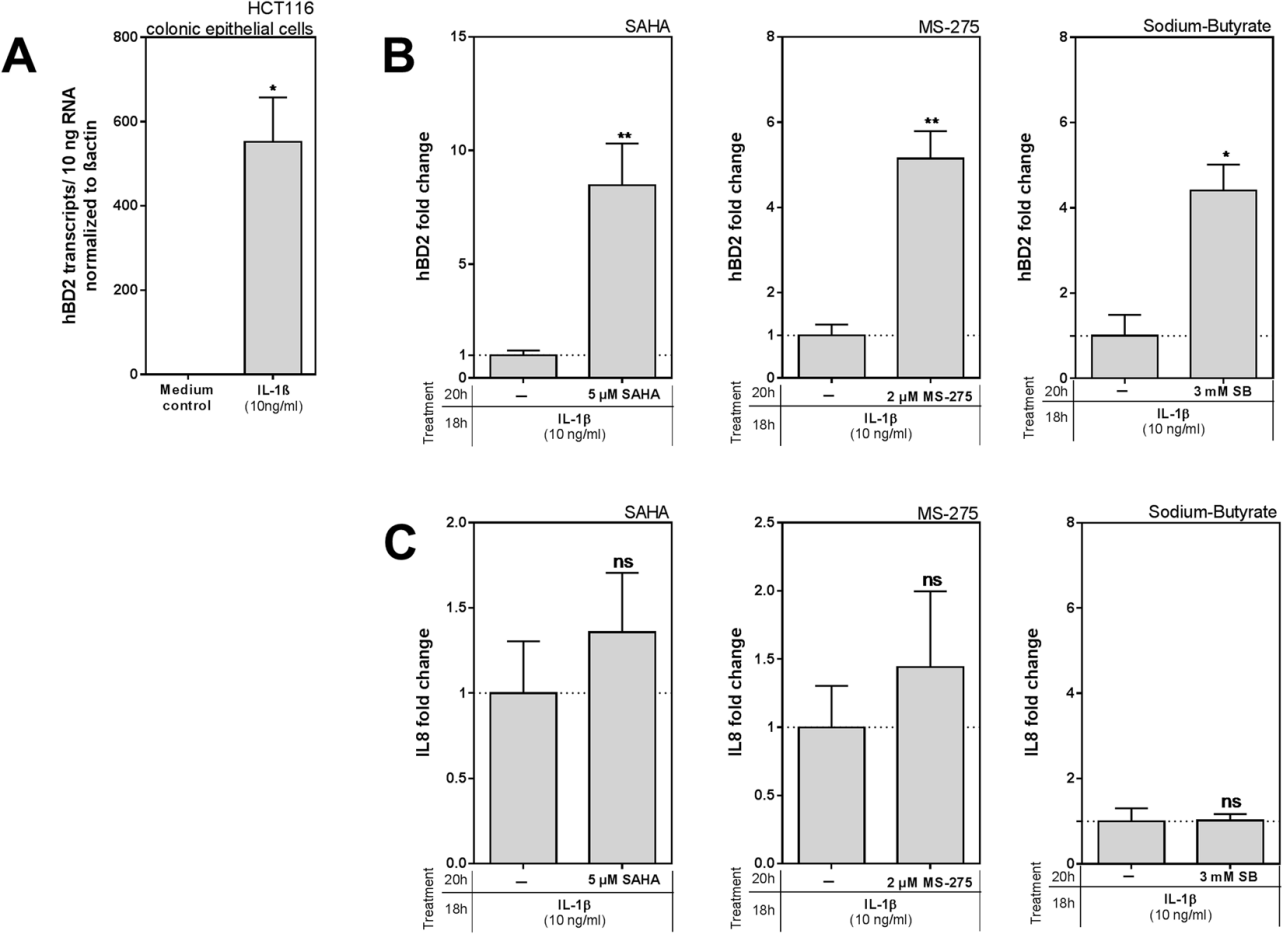
Interleukin 1β (IL1β)-mediated human β-defensin 2 (hBD2) induction is enhanced by histone deacetylase (HDAC) inhibition in HCT116 cells. (**A**) Induction level of hBD2 mRNA in HCT116 cells by IL1β (10 ng/ml) after 18 hrs of stimulation. Fold change in hBD2 (**B**) or IL8 (**C**) induction are shown compared to IL1β stimulation alone when co-treated with either SAHA (5 µM), MS-275 (2 µM), or SB (3 mM). Inhibitor treatment and stimulation were done exactly as for CaCo-2/TC7 cells. Depicted are results of at least three independent experiments carried out in biological triplicates. *p < 0.05, **p < 0.01, evaluated by Mann-Whitney *u* test.

Since this is the first description of IL1β-mediated hBD2 induction in the context of HDACi, we aimed to confirm our result in a second colonic epithelial cell line. Similar to the first cell line hBD2, was induced via IL1β in HCT116 cells, (Fig. 3A) and again the enhancing effect by HDACi could be observed (Fig. 3B). HDACi alone did not affect hBD2 induction but had an elevating effect on basal IL8 expression (data not shown). In contrast to the CaCo-2/TC7 cells however, IL1β-induced IL8 expression was not augmented when HDAC inhibitors were used (Fig. 3C) indicating a potential cell line specific inflammatory response.

### NF-κB dependency of the enhancement effect of HDAC inhibition on hBD2 induction

**Figure 4:**
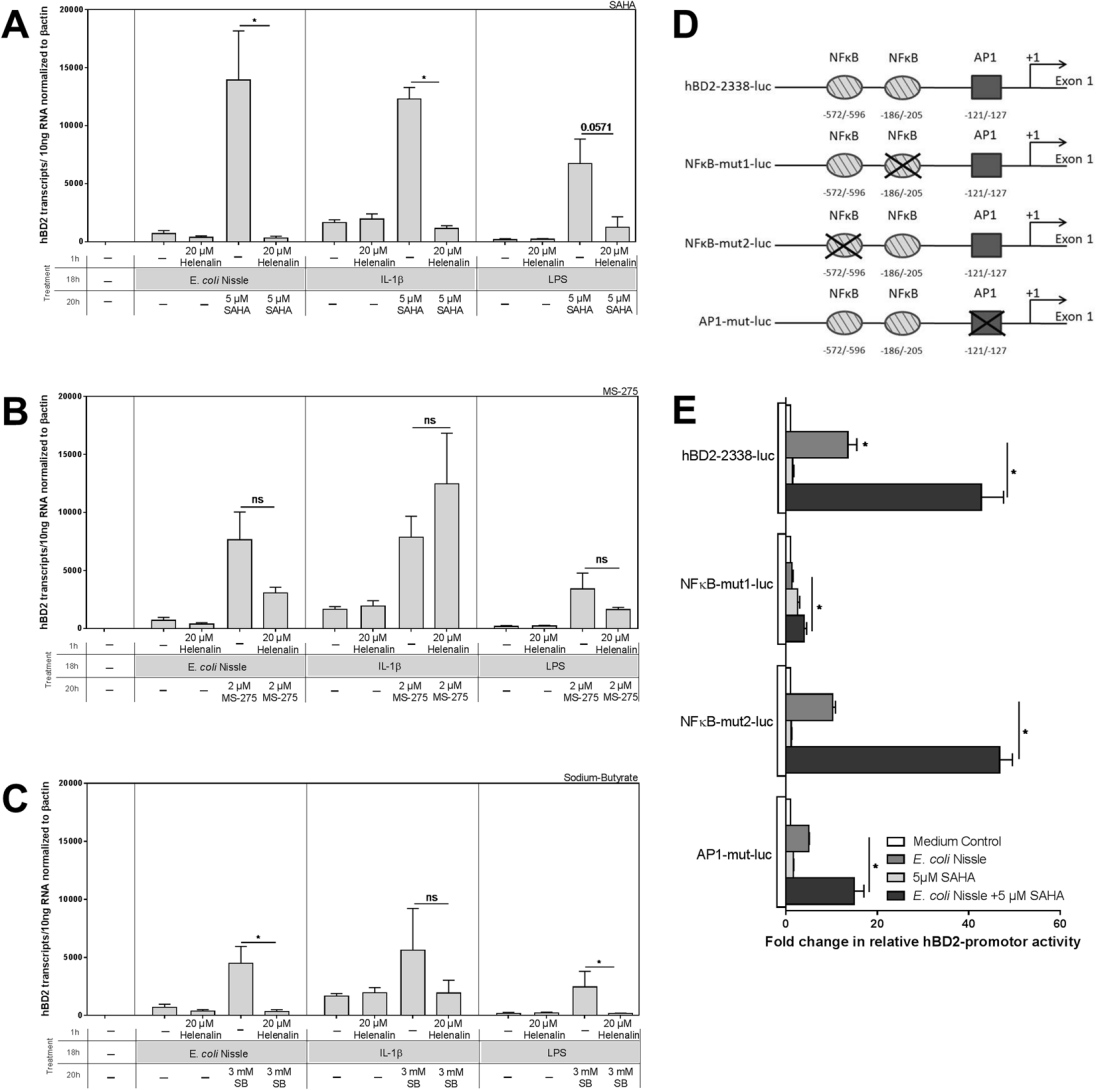
Pan-histone deacetylase (HDAC) inhibition enhancement of human β-defensin 2 (hBD2) expression is dependent on nuclear factor κ-light-chain-enhancer of activated B cells (NF-κB). (**A**–**C**) Show the abolishment of the enhancing effect of HDAC inhibition through Helenalin in CaCo-2/TC7 cells. HBD2 mRNA induction in response to EcN (3 × 10^8^ CFU/ml), IL1β (10 ng/ml) or LPS (1 µg/ml) alone, together with SAHA (5 µM) (**A**) MS-275 (2 µM) (**B**) or SB (3 mM) (**C**) and after pretreatment for 1 h with the NFkB inhibitor Helenalin (20 µM). Represented are the results of four independent experiments carried out in biological triplicates. Shown are relative copy numbers of hBD2 per 10 ng total RNA normalized to βactin expression. *p < 0.05 evaluated by Mann-Whitney *u* test. (**D**) Diagram of the used hBD2 promotor constructs (bp −2338 to −1 linked to the luciferase gene). Two NF-κB binding sites and one AP1 binding site are marked relative to the hBD-2 transcription start. Constructs with mutated binding sites were used as indicated. (**E**) Transfection of CaCo-2/TC7 cells took place with either the wild-type (hBD2-2338-luc) or the mutated hBD2 promotor constructs together with a Renilla luciferase plasmid as internal standard. 24 hrs post transfection, cells were treated with EcN (3 × 10^8^ CFU/ml) for 18 hrs, with SAHA (5 µM) for 20 hrs or a simultaneous combination of both (18 and 20 hrs, respectively). Promotor activation was measured subsequently and is displayed as luciferase activity normalized to Renilla activity. *p < 0.05 evaluated by Wilcoxon signed rank test.

In 2004, we could show that hBD2 induction via EcN utilizes at least two essential NF-κB binding sites in the promotor of hBD2^13^. The IL8 promotor also comprises binding sites for NF-kB^25^. Several recent studies have established a strong link between epigenetic control of NF-κB signaling and its transactivation function implicating a role for HDACs. It has been shown that HDAC1 and 2 interact with the p65 subunit to exert a corepressor function^26^ and that HDAC3- by deacetylating p65, facilitates the nuclear export of RelA which reduces NF-κB-dependent gene expression^3,27^. Inhibiting HDACs might therefore dampen their repressive effect on NFkB, leading to a more pronounced NFkB-dependent gene expression. So, based on these studies, we aimed to test the hypothesis whether the pronouncing effects of HDACi on hBD2 induction might indeed depend on NF-κB function. For this, we simultaneously inhibited NF-κB by Helenalin, which hinders DNA binding of p65 by alkylating it^28^, expecting it to avert the enhancement of hBD2 expression under HDACi. And indeed, the SAHA-mediated enhancement of hBD2 induction in response to all three stimulants was abolished (Fig. 4A). In the case of MS-275, blocking NF-κB only led to an attenuated effect when stimulation of hBD2 occurred via TLR activation (EcN or LPS) (Fig. 4B, left and right panel). For IL1β-induced hBD2, the MS-275-mediated enhancement was not blocked by NF-κB inhibition (Fig. 4B, middle panel), indicating that in that case, other transcription factors or signaling components activated by IL1β such as Activator protein 1 (AP1) or possibly Signal Transducers and Activators of Transcription (STATs)^29–31^ could come into play and mediate hBD2 upregulation. With SB, the picture looks more like the one with SAHA – blocking NF-κB in EcN or LPS-stimulated cells led to an abolishment of the HDAC-mediated enhancement (Fig. 4C, left and right panel). For IL1β, this trend was weaker but still present (Fig. 4C, middle panel). Taken together, these results demonstrate that NF-κB mediates the increase in hBD2 expression under pan-HDAC inhibition except for the combination of IL1β and the HDAC1 and 3 inhibitor MS-275 where other signaling mediators seem to be important.

To further verify the importance of NF-κB, we transfected CaCo-2/TC7 cells with different hBD2 promoter constructs containing mutations of either the proximal (positions −205 to −186) or the distal NF-κB binding sites (positions −596 to −572) and the AP1 binding site (positions −127 to −121; Fig. 4D). After treatment with SAHA and EcN, hBD2 promotor activation was analyzed (Fig. 4E). The wildtype construct (hBD2-2338-luc) showed an about 13 fold activation of the hBD2 promotor with EcN alone compared to untreated cells whilst together with HDACi this activation was augmented up to approximately 43 fold. Mutation of the proximal NF-κB binding site (NF-κB-mut1-luc) abolished EcN-induced hBD2 activation confirming a previous study from our group^13^ and reduced the EcN + SAHA-mediated potentiation effect to ~4 fold. Mutation of the distal binding site on the other hand did not show an effect on the inducibility of the promotor construct (hBD2-mut2-luc). Interestingly, the binding site for AP1 (AP1-mut-luc) seems to also play a role in not only the basal inducibility of the hBD2 promotor but also in reinforcing the amplitude of its activation, elevating it from ~5 fold (EcN) to ~14 fold. This effect is minor compared to NF-κB but significant. Collectively, these observations confirm the NF-κB involvement and show a role for AP1 in HDAC inhibition-mediated enhancement of hBD2 induction on the promotor level.

### *Ex vivo* investigation of the effect of HDAC inhibition on hBD2 inducibility

**Figure 5:**
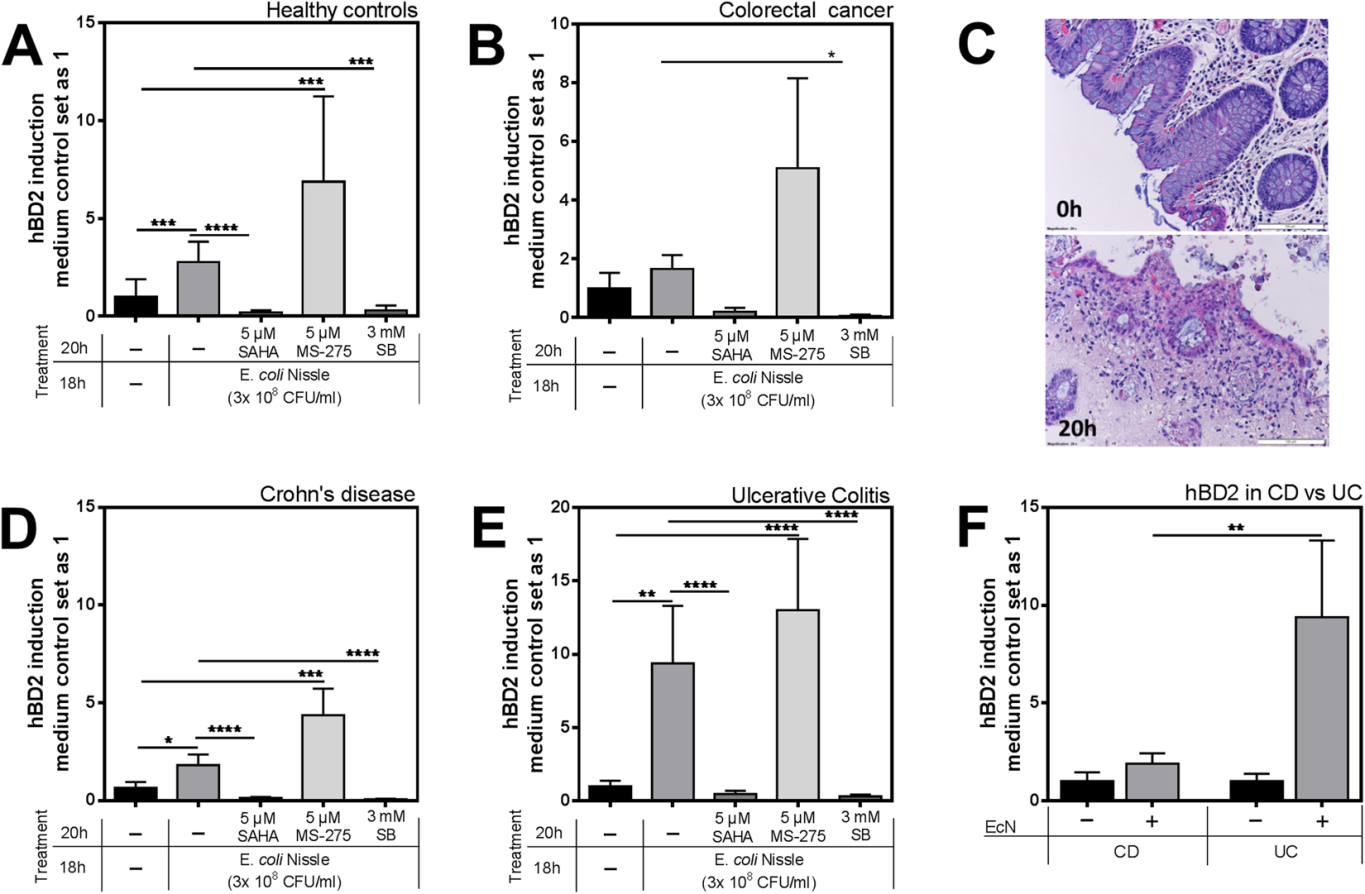
Inhibition of histone deacetylases (HDACs) hinders human β-defensin 2 (hBD2) induction in human colonic biopsies. HBD2 mRNA expression in cultured human colonic biopsies of (**A**) 13 healthy controls and (**B**) four colorectal tumor specimens in response to EcN, alone or together with either SAHA (5 µM), MS-275 (5 µM), or SB (3 mM). Inhibitor treatment started 2 hrs prior to the stimulation with EcN, which took then place in parallel to HDAC inhibition for another 18 hrs. (**C**) Hematoxylin and eosin staining of an uncultured (0 h) and medium cultured (20 h) healthy control biopsy. (**D**) HBD2 mRNA expression in colonic biopsies of 29 patients with Crohn’s Disease (CD) or 14 patients with ulcerative colitis (UC) (**E**). (**F**) Direct comparison of the hBD2 fold inductions in both disease entities CD or UC after EcN stimulation. Shown are relative fold changes compared to medium treatment alone according to 10 ng total RNA normalized to βactin expression. *p < 0.05, **p < 0.01, ***p < 0.001, ****p < 0.0001 evaluated by Mann-Whitney *u* test.

So far, most studies concerning the effect of HDAC inhibition on hBD2 inducibility have been conducted in mono-layered cell lines *in vitro*^18,19,32^. A major pitfall of using tumorous epithelial cell lines however is that HDACs are often overexpressed or generally deregulated in cancer^33–36^ potentially translating into differential responses that likely will not fully mirror the situation in healthy non-cancerous tissue. To be able to integrate the tissue context into our experiments, we established a human colonic biopsy culture, which is able to survive in a culture medium almost identical to the one used for the earlier CaCo-2/TC7 assays. Stimulation with the probiotic EcN and treatment with the HDAC inhibitors SAHA, MS-275 and SB was carried out exactly as in our *in vitro* setting. To also take the cancer aspect into consideration, we did not only examine mRNA expression of biospies from healthy controls (n = 13) (Fig. 5A), but also of biopsies taken from colorectal tumors (n = 4) (Fig. 5B). Medium controls of both healthy and tumorous biopsies showed a basal induction of hBD2 which has been set as one. Notably, we were able to strongly induce hBD2 with EcN treatment in *ex vivo* human colonic biopsies (Fig. 5A), contributing to a previous study, were we could show a significant *in vivo* increase of fecal hBD2 levels in healthy individuals after exposure to probiotics^12^. The induction of hBD2 in the tumor biopsies showed the same trend (Fig. 5B). Strikingly, inhibiting HDACs with SAHA or SB abolished hBD2 expression in both healthy and cancerous samples. However, inhibiting only HDACs 1 and 3 with MS-275 did not suffice to prevent hBD2 induction. Overall, it is important to note that healthy and tumorous tissue show a very similar response profile (Fig. 5A and B). Histochemical analyses using hematoxylin and eosin staining after cultivation shows that the tissue was affected to the extent that after 20 hrs it displayed a partial disintegration of the lamina propria as well as the epithelial lining (Fig. 5C). Investigation of viability via LDH showed no negative effects neither by EcN nor the different inhibitors (data not shown). Overall, cellular viability of cultured biopsies, seemed to still be in a good condition as determined by LDH assays (data not shown). Between the different treatments, no differences in tissue integrities were detected in histological stainings (data not shown).

In addition to healthy control and tumor biopsies, it was of interest to test whether the colonic mucosa of the two IBD entities CD or UC would display a different response pattern to HDACi. Also, we aimed to test if these two disease entities would demonstrate a disparity in their abilities to induce hBD2 being *ex vivo* stimulated with the potent inducer EcN. The basal induction of hBD2 that was found under medium treatment was defined as one to allow direct control. Interestingly, irrespective of the disease type, HDACi had the same effect and abolished EcN-mediated hBD2 mRNA expression in these patient samples (Fig. 5D and E). Most notably, colonic specimens from UC patients displayed a significantly stronger inducibility of hBD2 as compared to CD patients (Fig. 5F).

**Figure 6:**
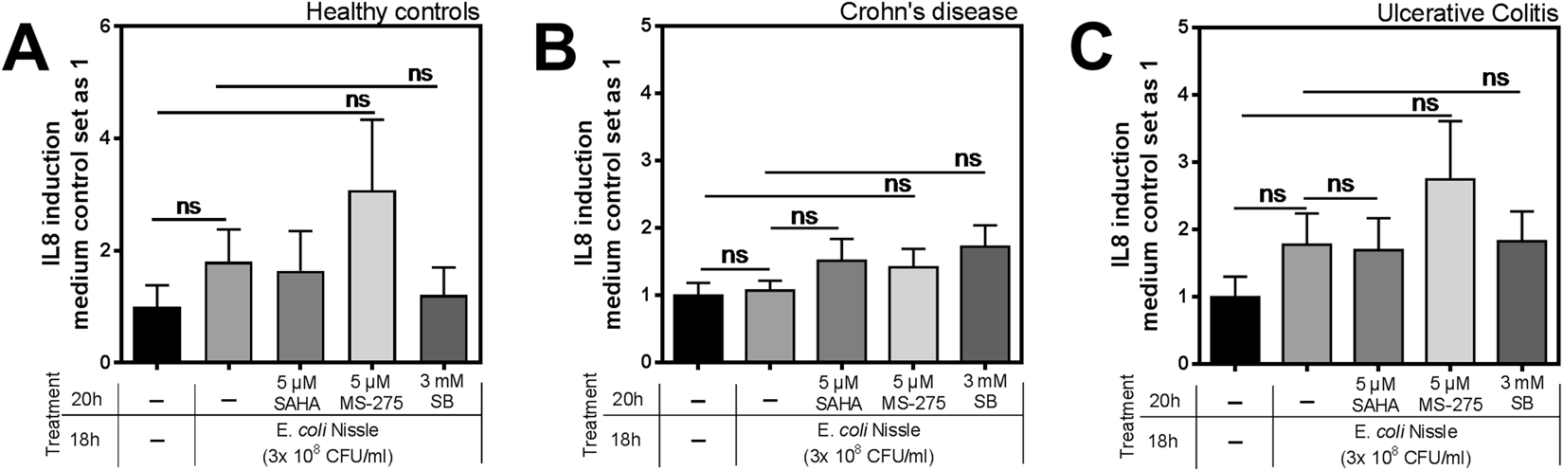
Effect of histone deacetylase (HDAC) inhibition on Interleukin 8 (IL8) expression in human colonic biopsies. IL8 expression in colonic biopsies of 13 healthy controls (**A**), 29 Crohn’s Disease (CD) (**B**), or 14 patients with ulcerative colitis (UC) (**C**) after treatment with EcN with or without simultaneous HDAC inhibition. Shown are relative fold changes compared to medium treatment alone according to 10 ng total RNA normalized to βactin expression. Data evaluated by Mann-Whitney *u* test.

We furthermore analyzed, whether IL8 expression was affected by HDACi in the same way as hBD2. We found that cultivation with medium alone led to an induction of IL8 in biopsies, but the different treatments did not further enhance this induction (Fig. 6). Comparable observations could be made for the expression levels of hBD1, which were not significantly altered by neither EcN alone nor the use of HDAC inhibitors (Fig. S2). Taken together, these results impressively demonstrate that EcN-mediated induction of hBD2 in a tissue compound is strongly dependent on HDAC function. This is true for healthy, tumorous, and tissue from IBD patients. These findings are in contrast to the *in vitro* findings on hBD2 regulation from cellular monolayers, which rather suggest a solely repressive effect of HDACs on the inducibility of hBD2.

## Discussion

The data here demonstrate a striking difference in functionality between different experimental settings. In line with and expanding previous investigations, we show a TLR-dependent hBD2 upregulation after HDAC inhibition in cell culture^13,17,19,37,38^. We furthermore demonstrated that this is also true for IL1β-mediated hBD2 induction in both, CaCo-2/TC7 as well as HCT116 cells. However, in contrast to a previous study^19^, we also observed an upregulation of IL8 in response to HDAC inhibition during our stimulation experiments in CaCo-2/TC7. The differing inflammatory responses regarding IL8 expression in CaCo-2/TC7 and HCT116 cells could likely be caused by differing epigenetic patterns in the two cell lines being owed to their cancerous nature^39^. This furthermore enhances the need for additional more *in vivo*-like experimental set ups. HDACs clearly modulate antimicrobial hBD2 expression but the effects differ dramatically between monolayer-cell versus tissue culture where hBD2 induction is blocked rather than enhanced.

This partial discrepancy might be owed to differences in study design. While Fischer *et al*. pretreated the cells with HDAC inhibitors overnight, before adding *E. coli* K12 for a short time^19^, we added the different stimulants 2 hrs after the inhibitors and incubated for an additional 18 hrs simultaneously. Consequently, our cell culture data with a simultaneous induction of IL8 do not support the suggestion that HDAC inhibition might serve as a pharmacological way to bolster epithelial antimicrobial defenses without the risk of an inflammatory response. Overall, it would therefore be interesting and of importance to look closer on the pharmacokinetics of HDACi in respect to antimicrobial and cytokine regulation. We furthermore cannot exclude that the effects found in *ex vivo*-treated biopsies might look different under different conditions and time points. However, the conclusion that HDACi could possibly have the potential to hinder hBD2 expression defies this limitation. The time points chosen in this study are based on the known kinetics for hBD2^12,13,38^ and reasonable durations for and concentrations of HDAC inhibition^24,35,40–43^.

While we have previously noted that NF-κB is a major regulator of hBD2 induction^13,19,37,38^, in the case of IL1β-mediated hBD2 expression other transcription factors might come into play. While a role of AP1 has been reported^13^, we cannot exclude further factors, including a putative feedback loop involving upregulated cytokines. This loop might involve IL8, but also the IL1β responsive IL6^31^ or others, that could affect STAT signaling-induced hBD2^29,30^. A strong NFκB dependency was mainly confirmed for TLR-mediated upregulation, but the basic effect of enhanced hBD2 induction after HDAC inhibition was seen in EcN and LPS, as well as IL1β treated cells.

The role of HDACs in hBD2 regulation consequently appears to be consistent for multiple inducers in the two *in vitro* systems studied, including different cell lines as well as cultured organoids^17,19^. However, analyzing this in human colonic tissue unveiled a completely opposite effect. When blocking all HDAC function, using either the pan-inhibitor SAHA or SB in human colonic biopsies, hBD2 upregulation by the probiotic EcN was completely abolished and expression levels returned to baseline. Only blocking HDACs 1 and 3 by using the more selective inhibitor MS-275 still allowed for EcN-mediated hBD2 induction but did not lead to the enhancement effect observed in cell culture. While SAHA or SB exposure had indeed an effect on the tissue response, we so far cannot exclude that the lack of MS-275 functionality could be due to limited uptake or other technical issues. Based on the available data, we hypothesize that the more selective inhibition of some HDACs cannot fully inhibit hBD2 induction in *ex vivo* treated biopsies. Future studies will have to clarify exactly which HDACs are responsible in preventing hBD2 induction *ex vivo*, or if all of them have to be blocked collectively. One limitation of the *ex vivo* biopsy approach is that we cannot rule out additional autocrine effects on hBD2 expression by pro-inflammatory mediators being released from the tissue itself. To be able to reduce this impact in our analyses, we are referring to fold changes in the EcN and HDACi treated biopsies. These are compared to the medium control samples which already express basal levels of hBD2.

The striking divergence from what we and others have found in cell culture allows to speculate that the role of HDACs in hBD2 regulation might be more complex, dynamic and context-dependent than previously thought. Especially since Fischer *et al*. saw comparable effects in cultured epithelial organoids^19^, other non-epithelial mucosal cell types or potentially even infiltrated immune cells might be involved in reversing the HDAC inhibitor effect on the epithelium. However, the mechanisms that underlie the divergence between tissue- and cell culture/organoids regarding hBD2 remain to be uncovered. Nonetheless, if *in vivo*, HDAC inhibition could indeed abolish the induction potential of this potent antimicrobial peptide in colonic epithelia, it might potentially compromise innate immune defenses. HBD2 has been particularly studied in the context of IBD, especially in ulcerative colitis (UC) and colonic Crohn’s Disease (CD)^44^. While UC is characterized by a potent upregulation during active inflammation, CD patients display an attenuated hBD2 induction which might leave them vulnerable to resident microbiota^15,45^. Those significant differences have been observed in different IBD cohorts described by different groups^46–48^ even though the mechanisms are still unclear. Since the gene copy number of hBD2 varies, we proposed reduced gene copies of the beta defensin cluster as one possible explanation in German and US patients^49^. However this association could not be found in another cohort^50^. In line with these previous investigations describing distinct expression levels, we now demonstrate that Crohn’s disease patient mucosa is compromised in inducing hBD2 using *ex vivo* EcN treated biopsies. Like some other antimicrobials, hBD2 also possesses immunomodulary properties. In psoriasis, excessive hBD2 levels are thought to be a disease driver and a recent study showed they can trigger activation of plasmacytoid dendritic cells by breaking tolerance to self-DNA^51^. However, in another context and tissue, hBD2 has been reported to exhibit anti-inflammatory effects by downregulating several pro-inflammatory cytokines in LPS-stimulated lung epithelial cells^52^. It is therefore important to continue studies on the function of HDACs on antimicrobial peptide and cytokine expression. Furthermore, a better understanding of the possible functional consequences of HDACi in the specific disease contexts and specific organs is needed. Such work could employ parallel treatment with HDACi and innate immune stimulants focusing on *ex vivo* cultures of additional tissues other than colonic epithelia, as well as animal studies. Without a doubt, such studies will aid in further understanding the potential of HDAC inhibitor use in inflammatory and/or infectious diseases. Identifying the exact mechanisms by which HDACs synergize with or sabotage different TLR stimulants and other immune mediators to control antimicrobials and cytokines, will be instrumental in translating these findings into potential future therapy development.

## Material and Methods

### Patient material

All patients and controls included in this study gave their written and informed consent after they were informed about the study purpose, sample procedure, and potential adjunctive risks. The study protocol was previously approved by the ethical committee of the University Hospital Tübingen, Germany and all experiments were conducted in accordance with the relevant guidelines and regulations. Biopsies from the sigmoidal colon of healthy controls, patients with Crohn’s disease, and patients with ulcerative colitis were sampled during routine colonoscopy at the Robert-Bosch Hospital, Stuttgart, Germany and the University Hospital Tübingen, Germany. Diagnosis was performed according to standard criteria including clinical, radiological, endoscopic, and histopathological results. Biopsies from colorectal tumors were obtained within 90 minutes after resection of the tumors at the Marienhospital, Stuttgart, or at the University Hospital Tübingen, Germany. The detailed numbers of patient biopsies used for different experiments are specified in the according figure legends and in Table S1.

### Cell culture

CaCo-2/TC7^53^ were kindly received from Oliver Burk, IKP Stuttgart, Germany, and HCT116 cells provided from Heiko van der Kuip, IKP Stuttgart, Germany, were cultured in Dulbecco’s modified eagle medium (DMEM) containing 10% (v/v) fetal cow serum (FCS), 1% (v/v) non-essential amino acids, 1% (v/v) sodium pyruvate and 1% (v/v) penicillin/streptomycin (Pen/Strep) (all from Life Technologies) at 37 °C with 5% CO_2_. Experiments were performed in FCS- and Pen/Strep-free medium in 12-well-plates on cellular monolayers at about 80–90% confluency. After a total treatment duration of 20 hrs mRNA and protein was subsequently analyzed.

### Biopsy culture

Before working with colonic biopsies, an *ex vivo* ileal biopsy culture for the study of AMP regulation has been established in our lab^20^. Freshly collected colonic biopsies were washed several times with ice-cold PBS containing 10% (v/v) Pen/Strep, immediately transferred into wells of a 24-well-plate with 1 ml of designated media consisting of DMEM containing 2% (v/v) Pen/Strep to minimize bacterial overgrowth and incubated at 37 °C with 5% CO_2_ for a total treatment duration of 20 hrs. Thereafter biopsies were put into RNAlater until RNA isolation and the supernatant was collected for subsequent quality control. The integrity of total RNA was controlled using the RNA 6000 Nano Assay (Agilent, USA). The viability of the samples was tested via the supernatants using LDH-ELISA (Roche, Switzerland) according to manufacturer’s protocol.

### Bacteria

*E. coli* Nissle 1917 (EcN) (Ardeypharm, Germany) has been heat-inactivated for all experiments as has been described before^13^. We used heat-inactivated bacteria, since it has been shown that they show the same effect as living EcN^13^. Briefly, EcN has been grown overnight in trypticase soy broth (TSB) medium at 37 °C under constant shaking. The next morning, 100 µl of the bacterial suspension were diluted in 10 ml of TSB medium to keep bacteria in a linear growth phase and grown under shaking conditions at 37 °C. After about 3 hrs EcN were heat killed in a water bath at 65 °C for 45 min and then diluted to a concentration of 3 × 10^8^ cells/ml in FCS-free and Pen/Strep-free DMEM cell culture medium.

### Treatment of cells and biopsies

Cells or biopsies were pretreated with suberoylanilide hydroxamid acid (SAHA; Vorinostat) (InvivoGen, USA) MS-275 (Entinostat; Selleckchem, USA) or sodium-butyrate (SB) (Sigma-Aldrich, Germany) for 2 hrs prior to the start of the stimulation with either heat-inactivated *E. coli* Nissle 1917 (Ardeypharm, Germany), IL1β (PeproTech, Germany) or LPS from *E. coli* serotype O111:B4 (Sigma-Aldrich, Germany) which took place in parallel to HDAC inhibition for additional 18 hrs adding up to a total of 20 hrs of treatment. Treatment with the NF-κB inhibitor Helenalin (Enzo Life Sciences, USA) took place for 1 h right at the beginning of the 20 hrs and was then removed to avoid cytotoxic effects.

### Transient transfection and promotor activity

CaCo-2/TC7 cells were transfected with 500 ng DNA using Turbofect (Thermo Fisher Scientific, USA) according to manufacturer’s protocol. HBD2 reporter plasmids used in these experiments (hBD2-2338-luc, NF-κB-mut1-luc, NF-κB-mut2-luc, AP1-mut-luc) have been described previously^8,13^. CMV-Renilla plasmid (Promega, USA) was used as an internal standard to which firefly luciferase activity was normalized. Promotorless pGL3basic firefly luciferase vector (Promega, USA) being the backbone of the aforementioned hBD2 reporters, was used as control. Used cultivation media was the above described DMEM used for cell culture but without FCS and Pen/Strep. 24 hrs post transfection, treatment with indicated media or agents was started as described above in “treatment of cells and biopsies”. After 20 hrs of treatment cells were lysed for measuring luciferase activity with the Dual Luciferase Reporter Assay (Promega, USA) using an Enspire PlateReader (Perkin Elmer, USA). Firefly luciferase signals were normalized to Renilla activities, the latter representing the transfection efficiency. Each transfection was performed in triplicates in three or more independent experiments.

### Histological staining

For the hematoxylin and eosin staining of biopsy sections, biopsies were either directly fixed with 4% formalin or after 20 hrs of treatment. Obtained FFPE tissue slides were used for standard staining procedures with hematoxylin (Merck, Germany) for 7 mins and eosin (Sigma-Aldrich, Germany) for 3 mins. Examination of the slides was carried out with an BX63 microscope (Olympus, Germany). Picture acquisition was done using a DP80 camera and the imaging software cellSens Dimension (both from Olympus, Germany).

### Protein quantification

To measure the amount of hBD2 protein in CaCo-2/TC7 cell lysates, an enzyme-linked immunosorbent *assay* (Human Beta-Defensin2 (hBD2) ELISA Kit; Phoenix Pharmaceuticals, USA) was performed according to the manufacturer’s protocol. For this, cells were pelleted at 4 °C and washed. Lysis of cells was achieved by adding whole protein lysis buffer containing a protein inhibitor cocktail (PIC) to the pellet, incubating for 30 mins on ice under occasional vortexing. After that, lysed cells were centrifuged at 4 °C/10000 g for 25 mins. Protein concentration was determined out of the supernatants using the Bradford method.

### RNA preparation and Real-Time PCR

RNA from cells and tissue was isolated using the Quick-RNA™ MiniPrep Kit and the Direct-zol™ RNA MiniPrep Kit respectively (ZymoResearch, USA) according to the manufacturer’s protocol. RNA was quantified with a NanoDrop and transcribed into cDNA using the iScript™ cDNA Synthesis Kit (Bio-Rad, USA). The amount cDNA according to 10 ng RNA was applied in all assays. The absolute mRNA quantification was done using SYBR Green I (Roche, Switzerland) and measured in a Roche LightCycler 480. Used primers are listed in Table 1. The absolute copy numbers were calculated from the Cp-Values according to a standard plasmid.

**Table 1:** Primers used in quantitative real-time PCR.

Gene product	Forward Primer (5′->3′)	Reverse Primer (5′->3′)
hBD2	ATC AGC CAT CAG GGT CTT GT	GAG ACC ACA GGT GCC AAT TT
IL8	ATG ACT TCC AAG CTG GCC GTG GC	TCT CAG CCC TCT TCA AAA ACT TC
hBD1	GGC CTC AGG TGG TAA CTT TCT	TTC TTC TGG TCA CTC CCA GC
βactin	GCC AAC CGC GAG AAG ATG A	CAT CAC GAT GCC AGT GGT A

### Statistical Analysis

Expression differences between groups were analyzed using t-tests. Fold changes, normalized to control treatment, were analyzed by Wilcoxon signed rank tests or Mann-Whitney tests as indicated in the corresponding figure legends. All results are displayed by mean values + SEM. Values of p < 0.05 were considered to be statistically significant. Data were analyzed using GraphPad Prism 6.

## Electronic supplementary material

Supplementary Information

## Data Availability

Full data will be available.

## References

[1] Lehrmann H, Pritchard LL, Harel-Bellan A (2002). Histone acetyltransferases and deacetylases in the control of cell proliferation and differentiation. Adv. Cancer Res..

[2] Mai A (2005). Histone deacetylation in epigenetics: An attractive target for anticancer therapy. Med. Res. Rev..

[3] Chen L, Fischle W, Verdin E, Greene WC (2001). Duration of Nuclear NF-κB Action Regulated by Reversible Acetylation. Science.

[4] Singh BN (2010). Nonhistone protein acetylation as cancer therapy targets. Expert Rev. Anticancer Ther..

[5] Felice C, Lewis A, Armuzzi A, Lindsay JO, Silver A (2015). Review article: selective histone deacetylase isoforms as potential therapeutic targets in inflammatory bowel diseases. Aliment. Pharmacol. Ther..

[6] Glauben R, Siegmund B (2011). Inhibition of Histone Deacetylases in Inflammatory Bowel Diseases. Mol. Med..

[7] Schröder JM, Harder J (1999). Human beta-defensin-2. Int. J. Biochem. Cell Biol..

[8] Harder J (2000). Mucoid Pseudomonas aeruginosa, TNF-alpha, and IL-1beta, but not IL-6, induce human beta-defensin-2 in respiratory epithelia. Am. J. Respir. Cell Mol. Biol..

[9] Duits LA, Ravensbergen B, Rademaker M, Hiemstra PS, Nibbering PH (2002). Expression of beta-defensin 1 and 2 mRNA by human monocytes, macrophages and dendritic cells. Immunology.

[10] Wah J (2006). Antimicrobial peptides are present in immune and host defense cells of the human respiratory and gastrointestinal tracts. Cell Tissue Res..

[11] Yin L (2010). Differential and coordinated expression of defensins and cytokines by gingival epithelial cells and dendritic cells in response to oral bacteria. BMC Immunol..

[12] Möndel M (2009). Probiotic E. coli treatment mediates antimicrobial human beta-defensin synthesis and fecal excretion in humans. Mucosal Immunol..

[13] Wehkamp J (2004). NF-kappaB- and AP-1-mediated induction of human beta defensin-2 in intestinal epithelial cells by Escherichia coli Nissle 1917: a novel effect of a probiotic bacterium. Infect. Immun..

[14] Mack DR (2011). Probiotics in Inflammatory Bowel Diseases and Associated Conditions. Nutrients.

[15] Wehkamp J (2003). Inducible and constitutive beta-defensins are differentially expressed in Crohn’s disease and ulcerative colitis. Inflamm. Bowel Dis..

[16] Wehkamp J, Koslowski M, Wang G, Stange EF (2008). Barrier dysfunction due to distinct defensin deficiencies in small intestinal and colonic Crohn’s disease. Mucosal Immunol..

[17] Yin L, Chung WO (2011). Epigenetic regulation of human?-defensin 2 and CC chemokine ligand 20 expression in gingival epithelial cells in response to oral bacteria. Mucosal Immunol..

[18] Schwab M (2008). The dietary histone deacetylase inhibitor sulforaphane induces human?-defensin-2 in intestinal epithelial cells. Immunology.

[19] Fischer N (2016). Histone deacetylase inhibition enhances antimicrobial peptide but not inflammatory cytokine expression upon bacterial challenge. Proc. Natl. Acad. Sci..

[20] Courth LF (2015). Crohn’s disease-derived monocytes fail to induce Paneth cell defensins. Proc. Natl. Acad. Sci. USA.

[21] Beckers T (2007). Distinct pharmacological properties of second generation HDAC inhibitors with the benzamide or hydroxamate head group. Int. J. Cancer.

[22] Hu E (2003). Identification of novel isoform-selective inhibitors within class I histone deacetylases. J. Pharmacol. Exp. Ther..

[23] Khan N (2008). Determination of the class and isoform selectivity of small-molecule histone deacetylase inhibitors. Biochem. J..

[24] Tatamiya T, Saito A, Sugawara T, Nakanishi O (2004). Isozyme-selective activity of the HDAC inhibitor MS-275. Cancer Res..

[25] Hoffmann E, Dittrich-Breiholz O, Holtmann H, Kracht M (2002). Multiple control of interleukin-8 gene expression. J. Leukoc. Biol..

[26] Ashburner BP, Westerheide SD, Baldwin AS (2001). Thep65 (RelA) Subunit of NF-κB Interacts with the Histone Deacetylase (HDAC) Corepressors HDAC1 and HDAC2 To Negatively Regulate Gene Expression. Mol. Cell. Biol..

[27] Huang B, Yang X-D, Lamb A, Chen L-F (2010). Posttranslational modifications of NF-κB: another layer of regulation for NF-κB signaling pathway. Cell. Signal..

[28] Lyß G, Knorre A, Schmidt TJ, Pahl HL, Merfort I (1998). The Anti-inflammatory Sesquiterpene Lactone Helenalin Inhibits the Transcription Factor NF-κB by Directly Targeting p65. J. Biol. Chem..

[29] Albanesi C (2007). IL-4 and IL-13 Negatively Regulate TNF-α- and IFN-γ-Induced β-Defensin Expression through STAT-6, Suppressor of Cytokine Signaling (SOCS)-1, and SOCS-3. J. Immunol..

[30] Kanda N, Watanabe S (2008). IL-12, IL-23, and IL-27 enhance human beta-defensin-2 production in human keratinocytes. Eur. J. Immunol..

[31] Vitkus SJ, Hanifin SA, McGee DW (1998). Factors affecting Caco-2 intestinal epithelial cell interleukin-6 secretion. In Vitro Cell. Dev. Biol. Anim..

[32] Lan C-CE (2011). High-Glucose Environment Inhibits p38MAPK Signaling and Reduces Human β-Defensin-3 Expression [corrected] in Keratinocytes. Mol. Med. Camb. Mass.

[33] Ishihama K (2007). Expression of HDAC1 and CBP/p300 in human colorectal carcinomas. J. Clin. Pathol..

[34] Marks P (2001). Histone deacetylases and cancer: causes and therapies. Nat. Rev. Cancer.

[35] Wilson AJ (2006). Histone Deacetylase 3 (HDAC3) and Other Class I HDACs Regulate Colon Cell Maturation and p21 Expression and Are Deregulated in Human Colon Cancer. J. Biol. Chem..

[36] Yang H (2014). Overexpression of histone deacetylases in cancer cells is controlled by interplay of transcription factors and epigenetic modulators. FASEB J..

[37] Schlee M (2007). Induction of human beta-defensin 2 by the probiotic Escherichia coli Nissle 1917 is mediated through flagellin. Infect. Immun..

[38] Schlee M (2008). Probiotic lactobacilli and VSL#3 induce enterocyte beta-defensin 2. Clin. Exp. Immunol..

[39] Ahmed D (2013). Epigenetic and genetic features of 24 colon cancer cell lines. Oncogenesis.

[40] Oger F (2010). Biological and biophysical properties of the histone deacetylase inhibitor suberoylanilide hydroxamic acid are affected by the presence of short alkyl groups on the phenyl ring. J. Med. Chem..

[41] Leoni F (2002). The antitumor histone deacetylase inhibitor suberoylanilide hydroxamic acid exhibits antiinflammatory properties via suppression of cytokines. Proc. Natl. Acad. Sci. USA.

[42] Butler LM (2002). The histone deacetylase inhibitor SAHA arrests cancer cell growth, up-regulates thioredoxin-binding protein-2, and down-regulates thioredoxin. Proc. Natl. Acad. Sci..

[43] Boffa LC, Vidali G, Mann RS, Allfrey VG (1978). Suppression of histone deacetylation *in vivo* and *in vitro* by sodium butyrate. J. Biol. Chem..

[44] Jäger S, Stange EF, Wehkamp J (2013). Inflammatory bowel disease: an impaired barrier disease. Langenbecks Arch. Surg. Dtsch. Ges. Für Chir..

[45] Nuding S, Fellermann K, Wehkamp J, Stange EF (2007). Reduced mucosal antimicrobial activity in Crohn’s disease of the colon. Gut.

[46] Wehkamp J (2002). Human beta-defensin 2 but not beta-defensin 1 is expressed preferentially in colonic mucosa of inflammatory bowel disease. Eur. J. Gastroenterol. Hepatol..

[47] Fahlgren A, Hammarström S, Danielsson Å, Hammarström M-L (2003). Increased expression of antimicrobial peptides and lysozyme in colonic epithelial cells of patients with ulcerative colitis. Clin. Exp. Immunol..

[48] Zilbauer, M. *et al*. Expression of Human Beta-Defensins in Children with Chronic Inflammatory Bowel Disease. *PLoS ONE* **5**, (2010). 10.1371/journal.pone.0015389 PMC2962650 21042595

[49] Fellermann K (2006). A chromosome 8 gene-cluster polymorphism with low human beta-defensin 2 gene copy number predisposes to Crohn disease of the colon. Am. J. Hum. Genet..

[50] Aldhous MC, Noble CL, Satsangi J (2009). Dysregulation of human beta-defensin-2 protein in inflammatory bowel disease. PloS One.

[51] Lande R (2015). Cationic antimicrobial peptides in psoriatic skin cooperate to break innate tolerance to self-DNA. Eur. J. Immunol..

[52] Donnarumma G (2007). Anti-inflammatory effects of moxifloxacin and human β-defensin 2 association in human lung epithelial cell line (A549) stimulated with lipopolysaccharide. Peptides.

[53] Chantret I (1994). Differential expression of sucrase-isomaltase in clones isolated from early and late passages of the cell line Caco-2: evidence for glucose-dependent negative regulation. J. Cell Sci..

