# Supplementary material for: Histone deacetylase-mediated regulation of the antimicrobial peptide hBD2 differs in intestinal cell lines and cultured tissue
Source: Sci Rep. 2018 Aug 27;8:12886. doi: 10.1038/s41598-018-31125-x (PMC6110836; doi:10.1038/s41598-018-31125-x)
Supplement: Supplementary file 1 — Supplementary Information [file 41598_2018_31125_MOESM1_ESM.pdf]

# Histone deacetylase-mediated regulation of the antimicrobial peptide hBD2 differs in intestinal cell lines and cultured tissue

Sabrina Stebe-Frick[1§], Maureen J. Ostaff[2#§], Eduard F. Stange[1], Nisar P. Malek[1], and Jan Wehkamp[1*]

**Histone deacetylase-mediated regulation of the antimicrobial peptide hBD2 differs in intestinal cell lines and cultured tissue**

Sabrina Stebe-Frick[1§], Maureen J. Ostaff[2#§], Eduard F. Stange[1], Nisar P. Malek[1], and Jan Wehkamp[1*]

## Supplementary Table 1

### *Ex vivo* colonic biopsy cohort

| Group | N [total] | Gender | | Age [mean±SEM] |
|---|---|---|---|---|
| | | f | m | |
| CD Patients | 29 | 38 % | 62 % | 43 ± 2.36 |
| UC Patients | 14 | 36 % | 64 % | 39.92 ± 2.7 |
| Controls | 13 | 53 % | 47 % | 53.69 ± 3.4 |
| Colonic carcinoma | 4 | 25 % | 75 % | 66.33 ± 9.5 |

**Histone deacetylase-mediated regulation of the antimicrobial peptide hBD2 differs in intestinal cell lines and cultured tissue**

Sabrina Stebe-Frick1§, Maureen J. Ostaff2#§, Eduard F. Stange1, Nisar P. Malek1, and Jan Wehkamp1*

# Suppl. Fig. 1

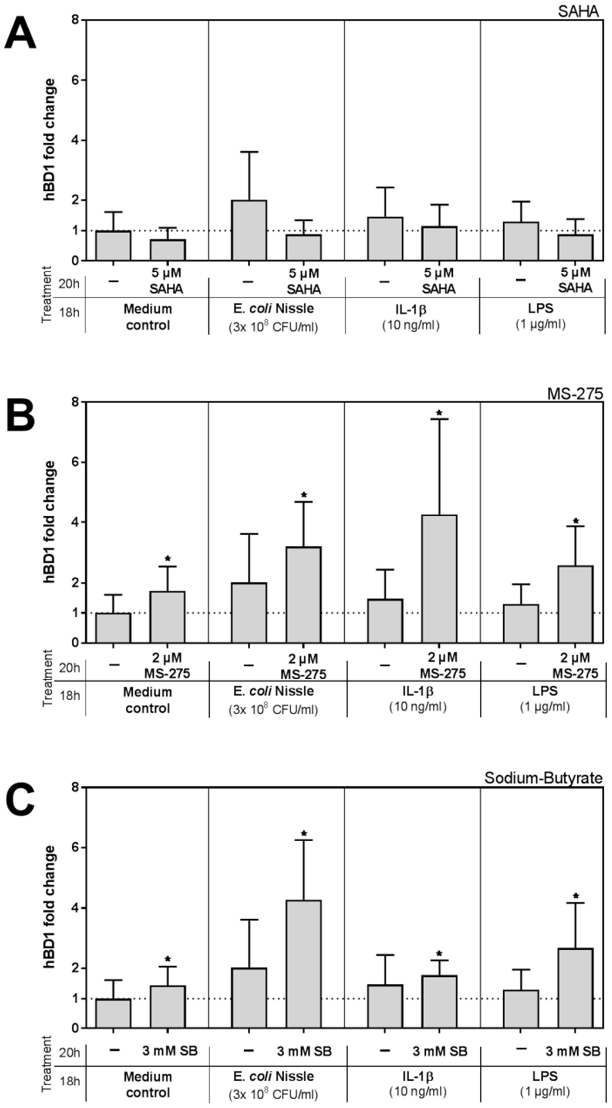


**Fig. S1** The effect of histone deacetylase (HDAC) inhibition on human ß-defensin 1 (hBD1) expression *in vitro*.

Fold changes in relative HBD1 mRNA expression after (A) treatment with HDAC inhibitor SAHA (5 µM), (B) MS-275 (2 µM) or (C) SB (3 mM) each for 20hrs together with or without the probiotic strain E. coli Nissle 1917 (3 × 108 CFU/ml), IL1ß (10 ng/ml) or LPS (1 µg/ml) each for 18 hrs. Expression of hBD1 according to 10 ng total RNA normalized to ßactin expression. Inhibitor treatment started 2 hrs prior to the stimulations, which took then place in parallel to HDAC inhibition for another 18 hrs. Shown are results of at least three independent experiments carried out in biological triplicates. * p<0.05, ** p<0.01evaluated by Mann-Whitney u test.

**Histone deacetylase-mediated regulation of the antimicrobial peptide hBD2 differs in intestinal cell lines and cultured tissue**

Sabrina Stebe-Frick1§, Maureen J. Ostaff2#§, Eduard F. Stange1, Nisar P. Malek1, and Jan Wehkamp1*

# Suppl. Fig. 2

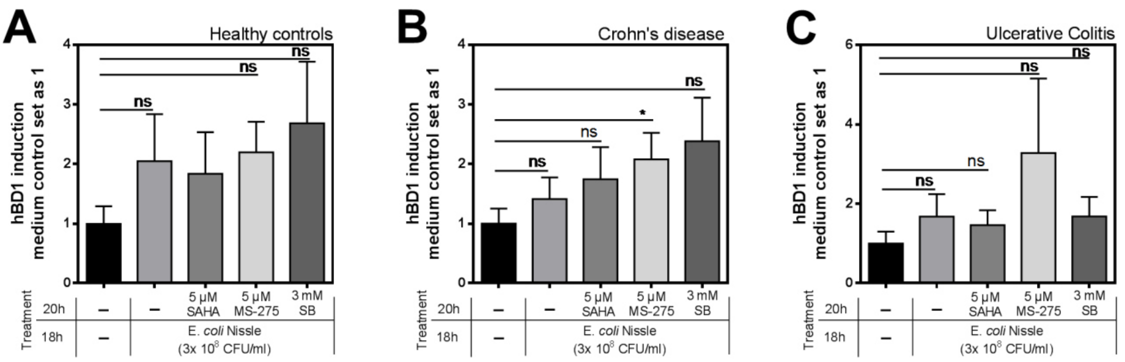


Fig. S2 The effect of histone deacetylase (HDAC) inhibition on human ß-defensin 1 (hBD1) expression in cultured human biopsies.

HBD1 mRNA expression in cultured human colonic biopsies of 13 healthy controls (A), 29 Crohn's disease patients (B), and 15 patients with ulcerative colitis (C) in response to EcN, alone or together with either SAHA (5 µM), MS-275 (2 µM), or SB (3 mM). Inhibitor treatment started 2 hrs prior to the stimulation with EcN, which took then place in parallel to HDAC inhibition for another 18 hrs. Shown are relative fold changes compared to medium treatment alone according to 10 ng total RNA normalized to ßactin expression.
